# The Role of Follistatin-like 1 in the Cross-Talk Among Osteoclastogenesis, Bone Marrow Stromal Cell Migration, and Osteoblastogenesis *In Vitro*

**DOI:** 10.3390/biomedicines14030555

**Published:** 2026-02-28

**Authors:** Yongxu Piao, Xiangguo Che, Xian Jin, Dong-Kyo Lee, Min Park, Eun-Jung Heo, Jinyoung Oh, Seong-Gon Kim, Dae-Chul Cho, Hyun-Ju Kim, Je-Yong Choi

**Affiliations:** 1 Department of Dentistry, Jiangsu Vocational College of Medicine, Yancheng 224005, China; 2 Department of Biochemistry and Cell Biology, Cell and Matrix Research Institute, Skeletal Disease Center, School of Medicine, Kyungpook National University, Daegu 41944, Republic of Korea; xiangguo0622@naver.com (X.C.); kimhan911021@naver.com (X.J.); ehdehd7710@naver.com (D.-K.L.); andrew990625@naver.com (M.P.); biohjk@knu.ac.kr (H.-J.K.); 3 Department of Anesthesiology and Pain Medicine, School of Medicine, Kyungpook National University, Daegu 41944, Republic of Korea; ahmeva0301@gmail.com (E.-J.H.); sgcms3@gmail.com (J.O.); 4 Department of Oral and Maxillofacial Surgery, College of Dentistry, Gangneung-Wonju National University, 7 Jukheon-gil, Gangneung-si 25457, Republic of Korea; kimsg@gwnu.ac.kr; 5 Department of Neurosurgery, School of Medicine, Kyungpook National University, Kyungpook National University Hospital, 130 Dongduk-Ro, Jung-Gu, Daegu 41944, Republic of Korea; dccho@knu.ac.kr

**Keywords:** Follistatin-like 1, bone remodeling, osteoclast differentiation, mesenchymal stem cell, chemotaxis, osteoblast differentiation, osteoporosis therapy

## Abstract

**Background**: Bone remodeling depends on the dynamic balance between osteoclast-mediated bone resorption and osteoblast-mediated bone formation. Follistatin-like 1 (FSTL1) has been reported as an osteoclast-secreted protein that inhibits osteoclast differentiation, but its direct effects on osteoblast differentiation remain unclear. This study aimed to determine whether FSTL1 regulates osteoblast differentiation and mesenchymal stem cell migration and characterizes its role in osteoclast-osteoblast cellular cross-talk under *in vitro* conditions. **Methods**: Bone marrow-derived macrophages (BMMs) and stromal cells (BMSCs) from mice were used to induce osteoclast and osteoblast differentiation, respectively. Chemotaxis was assessed by Transwell migration, and osteoblast differentiation was evaluated in BMSC and MC3T3-E1 cells using staining, qRT-PCR, Western blotting, and proliferation assays. **Results**: FSTL1 significantly suppressed osteoclast differentiation and resorptive activity, confirmed by TRAP staining and pit assay, respectively. Expression of osteoclast markers such as NFATc1, TRAP, and DC-STAMP was reduced under FSTL1 treatment. In BMSCs, FSTL1 did not affect proliferation but significantly enhanced chemotaxis. Moreover, FSTL1 promoted osteogenic differentiation and mineralization, as demonstrated by increased ALP activity and Alizarin Red S staining. In MC3T3-E1 pre-osteoblasts, FSTL1 increased cell proliferation and mineralization by MTS and Alizarin Red staining. Key osteogenic markers, including Runx2 and osteocalcin, were also upregulated. **Conclusions**: Osteoclast-derived FSTL1 significantly suppresses osteoclastogenesis and promotes mesenchymal cell chemotaxis and osteogenic differentiation, indicating a role in regulating osteoclast–osteoblast cellular interactions *in vitro*. Targeting FSTL1 signaling may represent a promising therapeutic strategy for osteoporosis and other disorders of impaired bone remodeling.

## 1. Introduction

Bone remodeling is a highly coordinated and continuous process involving the sequential actions of bone resorption by osteoclasts and bone formation by osteoblasts [1,2,3,4]. This dynamic turnover replaces old or damaged bone tissues with new bone, thereby maintaining structural integrity, mineral homeostasis, and adaptation to mechanical loading. Proper coupling between osteoclast-mediated resorption and osteoblast-mediated formation is essential for preserving bone mass and quality. Disruption of this balance underlies the pathogenesis of several bone disorders, such as osteoporosis, which is characterized by reduced bone mass, compromised microarchitecture, and increased fracture risk [5,6].

Osteoblasts and osteoclasts communicate through an integrated signaling network of paracrine factors, extracellular vesicles, cytokines, and matrix-derived molecules [7,8,9,10,11]. Several osteoclast-derived molecules, including collagen triple helix repeat containing 1 (Cthrc1), platelet-derived growth factor-BB (PDGF-BB), semaphorin 4D (Sema4D), and bone morphogenetic protein 6 (BMP6), have been identified as coupling factors that regulate mesenchymal cell recruitment, osteoblast differentiation, and matrix deposition [3,12,13,14,15]. For example, Cthrc1 and PDGF-BB promote mesenchymal stem cell migration and osteogenesis [12,14], while soluble Sema4D inhibits IGF-1-dependent osteogenesis and enhances osteoclast-driven bone resorption [16]. In addition, osteoclast-derived exosomes containing miRNAs such as miR-214 and miR-24-2-5p have been shown to modulate osteoblast lineage commitment and function that regulate osteoblast lineage commitment and functional activity [11,17]. Despite these advances, the molecular events coordinating the transition from resorption to formation remain incompletely defined, and novel coupling factors continue to be discovered. Molecules capable of concurrently inhibiting osteoclast activity and stimulating osteoblast differentiation are especially valuable, as dual-function regulators hold strong therapeutic potential for osteoporosis.

Follistatin-like 1 (FSTL1), also known as TSC-36, is a secreted glycoprotein originally identified as a TGF-β inducible protein. FSTL1 is broadly expressed across tissues and implicated in inflammation, tissue repair, immune modulation, and fibrosis. Recent studies highlight its roles in cardiovascular remodeling, chondrogenesis, and skeletal development [18,19]. FSTL1 is critical for maintaining articular cartilage homeostasis, with reduced expression driving terminal chondrocyte differentiation and matrix disruption, whereas restoration of FSTL1 enhances COL2A1 expression; notably, FSTL1 levels are decreased in human osteoarthritic cartilage [20]. However, its effects on osteoblast differentiation, mineralization, mesenchymal stromal cell behavior, and bone formation remain largely unexplored. FSTL1 has been identified by its secretion from osteoclasts [21]; however, the functional roles of FSTL1 in osteoclastogenesis and osteogenesis remain controversial and incompletely defined. Here, we propose that FSTL1 as a dual regulator regulates both osteoclast and osteoblast lineage activities and may contribute to osteoclast–osteoblast cellular cross-talk during bone remodeling under *in vitro* conditions.

In this study, we investigated the effects of recombinant FSTL1 on osteoblast precursor cells and primary BMSCs *in vitro*. By evaluating proliferation, migration, mineralization, and the expression of osteogenic markers, we aimed to determine whether FSTL1 regulates osteoblast lineage activity and mesenchymal stem cell behavior. Our findings demonstrate that FSTL1 enhances mesenchymal cell migration and osteogenic differentiation under *in vitro* conditions, suggesting that it plays a role in modulating osteoclast–osteoblast cellular interactions. Further *in vivo* studies are required to define its physiological significance and therapeutic potential in bone remodeling and osteoporosis.

## 2. Materials and Methods

### 2.1. Antibodies and Reagents

Alizarin Red S (ARS), alkaline phosphatase (ALP) staining kit, ascorbic acid, benzoyl peroxide, dimethyl sulfoxide (DMSO), ethanol, Fast Red Violet LB, glycerol, naphthol AS-MX phosphate, N,N-dimethylformamide, nitric acid, nonylphenyl-polyethyleneglycol acetate, paraformaldehyde (PFA), picric acid solution, silver nitrate, sodium acetate, sodium carbonate, sodium thiosulfate, β-glycerophosphate, and 2-methoxyethyl acetate (AME) were purchased from Sigma (St. Louis, MO, USA). Acetone, formaldehyde solution, sodium acetate trihydrate, and sodium tartrate dihydrate were obtained from JUNSEI (Tokyo, Japan). α-Minimum Essential Medium (α-MEM) and Permount were obtained from Thermo Scientific (Rockford, IL, USA). Fetal bovine serum (FBS) was obtained from GIBCO (Grand Island, NY, USA). Mouse M-CSF was purchased from PeproTech (Rocky Hill, NJ, USA). Methyl methacrylate (MMA) was obtained from Merck KGaA (Darmstadt, Germany), and N,N-dimethyl-p-toluidine was obtained from MP Biomedicals (Solon, OH, USA). Oligo(dT) primers were purchased from Promega (Madison, WI, USA). Penicillin/streptomycin was obtained from Lonza (Rockland, ME, USA). Red blood cell lysis buffer was obtained from BioLegend (San Diego, CA, USA). Recombinant FSTL1, the receptor activator of nuclear factor κB ligand (RANKL), and macrophage colony stimulating factor (M-CSF) were obtained from R&D Systems (Minneapolis, MN, USA). SuperScript II reverse transcriptase was obtained from iNtRON Biotechnology (Gyeonggi-do, Korea). SYBR Green master mix was obtained from ABI (Carlsbad, CA, USA).

### 2.2. Animals and Cell Culture

C57BL/6N female mice were purchased from KOATECH (Gyeonggi-do, Republic of Korea) and used for the isolation of bone marrow-derived macrophages (BMMs) and bone marrow-derived stromal cells (BMSCs). All animal care and experimental procedures were approved by the Institutional Animal Care and Use Committee of Kyungpook National University (KNU 2021-0101). Mice were housed in groups of fewer than five per cage under specific pathogen-free conditions, maintained on a 12 h light/dark cycle at a temperature of 22 ± 2 °C, and provided with standard rodent chow and water ad libitum.

Mouse BMMs were prepared from the femurs and tibias of 8-week-old male mice as previously described [22]. To assess FSTL1’s effect on osteoclastogenesis, BMMs were seeded at a density of 1 × 10^4^ cells per well in 96-well culture plates and cultured in α-MEM supplemented with 10% fetal bovine serum (FBS), penicillin/streptomycin, M-CSF (30 ng/mL), and receptor activator of nuclear factor-κB ligand (RANKL, 30 ng/mL). FSTL1 was administered at various concentrations (0, 10, and 100 ng/mL) on days 1 and 3 during osteoclast differentiation.

BMSCs were isolated from the long bones of 8-week-old male mice. Bone marrow cells were flushed, plated for 12 h, and non-adherent cells were removed. The remaining adherent cells were expanded in α-MEM supplemented with 10% fetal bovine serum (FBS), and passage 3 cells were used for migration assays and osteogenic differentiation.

MC3T3-E1 cells (mouse calvaria-derived pre-osteoblast line) were cultured in α-MEM supplemented with 10% FBS, 100 U/mL of penicillin, and 100 U/mL of streptomycin at 37 °C in a humidified atmosphere containing 5% CO_2_, as described in a previous study. For osteogenic differentiation, BMSC or MC3T3-E1 cells were seeded at 3 × 10^4^ cells/well in a 48-well plate. At confluence, cells were incubated in osteogenic medium containing ascorbic acid (50 μg/mL) and β-glycerophosphate (10 mM), with FSTL1 at various concentrations (0, 10, and 100 ng/mL). The differentiation medium was replaced every 3 days [23].

### 2.3. Cell Proliferation Assay (MTS)

Cell proliferation was evaluated by MTS assay. Cells were seeded at 5 × 10^3^ cells/well in 96-well plates, allowed to attach for 24 h, and treated with FSTL1 (0, 10, or 100 ng/mL) for 24 h. MTS solution (10 μL) was added to 100 μL of medium per well and incubated for 4 h. After shaking, the absorbance was measured at 590 nm using an ELISA reader.

### 2.4. Migration Assay

BMSC migration was evaluated using Transwell chambers (Corning, NY, USA). Cells (5 × 10^4^/well) were seeded in the upper chamber with serum-free medium. The lower chamber contained 1.5 mL of α-MEM with 10% FBS and FSTL1 (0, 10, or 100 ng/mL). After 24 h, migrated cells on the lower surface were fixed with 4% paraformaldehyde (PFA, Junsei, Tokyo, Japan) for 5 min at room temperature (RT), washed 3 times with phosphate-buffered saline (PBS), and stained with 0.1% crystal violet (Sigma-Aldrich, 3050 Spruce St., Saint Louis, MO, USA) for 30 min at RT. Non-migrated cells on the upper surface were gently removed using cotton swabs. Images of migrated cells were captured using a light microscope (Leica DMI4000 B), and migrated cells were quantified using ImageJ software.

### 2.5. Tartrate-Resistant Acid Phosphatase (TRAP) Staining

Osteoclasts differentiated for 3 days were fixed with 4%PFA for 1 min, followed by refixation with an ethanol/acetone solution (50%/50%) for 45 s, and then air-dried completely. Cells were then incubated with TRAP staining solution containing 0.1 mg/mL of naphthol AS-MX phosphate, 0.3 mg/mL of Fast Red Violet, and 0.83% N,N-dimethylformamide for 20 min at RT, as shown in a previous study [24].

### 2.6. Pit Assay

Osteoclast resorption assays were performed using commercial dentine disks (Os-teoSite, IDS, Tyne & Wear, UK). The disks were 5 mm in diameter and nominal thickness, 0.3 mm, and used without additional preparation other than pre-equilibration in culture medium prior to cell seeding. BMMs were seeded onto the dentine disks and cultured under osteoclastogenic conditions for resorption analysis. Cells were removed from the bone slices using cotton swabs, and the slices were incubated with peroxidase-conjugated wheat germ agglutinin (Sigma-Aldrich), followed by staining with 3,3′-diaminobenzidine (DAB, Sigma-Aldrich) on day 7 of osteoclast differentiation. The osteoclast resorption area was quantified using Bioquant Osteo 2019 software (v19.9.60; Bioquant Osteo, Nashville, TN, USA).

### 2.7. Alkaline Phosphatase (ALP) Staining

For ALP staining, differentiated osteoblasts were gently washed twice with PBS and fixed with 4% PFA for 15 min at RT on day 7 of osteogenic differentiation. After fixation, the cells were rinsed with PBS 3 times and incubated with an ALP staining solution (Sigma-Aldrich) according to the manufacturer’s instructions. The ALP staining intensity was quantified by the Bioquant Osteo 2019 software.

### 2.8. Alizarin Red S (ARS) Staining

Osteoblast mineralization was detected by ARS staining. Osteoblasts differentiated for 28 days were washed three times with PBS, fixed with 4% PFA for 10 min at RT, and stained with 2% alizarin red (pH 4.2) to evaluate calcium deposition. Cells were washed with deionized water, image was captured by Leica microscope, and ARS intensity was quantified by the Bioquant Osteo 2019 v19.9.60 program [25].

### 2.9. Western Blotting

Total protein was extracted from cells using M-PER™ Mammalian Protein Extraction Reagent (Thermo Fisher Scientific, Waltham, MA, USA) supplemented with a cocktail of protease inhibitors and phosphatase inhibitors. Protein concentrations were determined using a Bradford protein assay kit (Bio-Rad, Hercules, CA, USA). Equal amounts (20 μg) were loaded and separated on 10% SDS–polyacrylamide gels and then transferred onto polyvinylidene difluoride (PVDF) membranes. Membranes were blocked with 5% skim milk for 1 h at RT and incubated overnight at 4 °C with primary antibodies and then with horseradish peroxidase (HRP)-conjugated secondary antibodies for 1 h after washing with Tris-buffered saline containing 1% Tween-20 (TBST). Signals were detected using an enhanced chemiluminescence (ECL) detection system (GE Healthcare, Chicago, IL, USA) [26].

### 2.10. qRT-PCR

Total RNA was isolated using the easy-BLUETM Kit (iNtRON Biotechnology, Seongnam-si, Gyeonggi-do, Korea). RNA was separated using bromochloropropane (BCP, Sigma-Aldrich), precipitated with isoamyl alcohol (Sigma-Aldrich), washed with 70% ethanol, air-dried, and dissolved in nuclease-free water. Complementary DNA (cDNA) was synthesized from 1 μg of total RNA using SuperScript™ II reverse transcriptase (Invitrogen, CA, USA). Primer sequences for qRT-PCR were designed using NCBI’s Primer-BLAST (https://www.ncbi.nlm.nih.gov/tools/primer-blast/, accessed on 23 February 2026), and the sequences are listed in Table 1. qRT-PCR was performed using the Power SYBR green master mixture (Applied Biosystems, Foster, CA, USA).

### 2.11. Statistical Analysis

Statistical analyses were performed using GraphPad Prism 9 (GraphPad Software, San Diego, CA, USA). Data are presented as mean ± standard deviation (SD). For comparisons between groups, Student’s *t*-test was utilized to assess the significance of differences in means. Statistical significance was defined as *p* < 0.05 (*) and *p* < 0.01 (**).

## 3. Results

### 3.1. Effect of FSTL1 on Osteoclastogenesis

**Figure 1:**
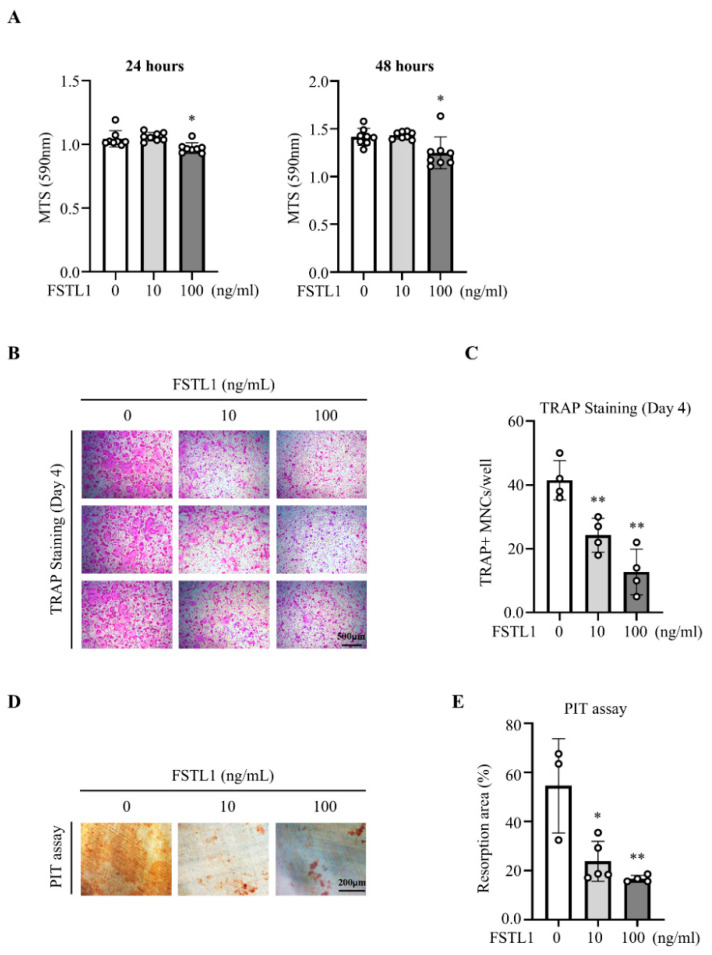
FSTL1 inhibited osteoclast differentiation and resorption activity. (**A**) BMMs were seeded at 5 × 10^3^ cells/well in 96-well plates, treated with FSTL1 (0, 10, or 100 ng/mL), and proliferation was measured at 24 and 48 h using the MTS assay (*n* = 8) (*n* = 4/group). (**B**) Osteoclast differentiation was evaluated by TRAP staining on day 4 of osteoclastogenic induction (*n* = 4/group). (**C**) Representative TRAP-stained images were obtained by light microscopy, and TRAP-positive multinucleated cells (MNCs) were quantified using Bioquant Osteo 2019 (v19.9.60) (*n* = 4/group). (**D**) Osteoclast resorption activity was assessed by the pit formation assay on day 4. FSTL1-0 ng/mL (*n* = 3), FSTL1-10 ng/mL (*n* = 4), and FSTL1-100 ng/mL (*n* = 5). (**E**) Resorption areas were quantified by using Bioquant Osteo 2019 (v19.9.60). * *p* < 0.05, ** *p* < 0.01 compared with the control group.

Bone marrow-derived macrophages (BMMs) were treated with recombinant mouse FSTL1 (rmFSTL1) at concentrations of 0, 10, and 100 ng/mL. MTS assay revealed that proliferation was significantly reduced at 100 ng/mL but not at 10 ng/mL (Figure 1A). During RANKL-induced differentiation, TRAP staining showed that rmFSTL1 markedly suppressed osteoblast formation at both concentrations (Figure 1B,C). Consistent with these findings, pit formation assays demonstrated that rmFSTL1 significantly reduced resorptive activity (Figure 1D,E). Together, these results identify FSTL1 as a strong inhibitor of osteoclastogenesis and bone resorption.

### 3.2. Downregulation of Osteoclastogenic Markers by FSTL1

**Figure 2:**
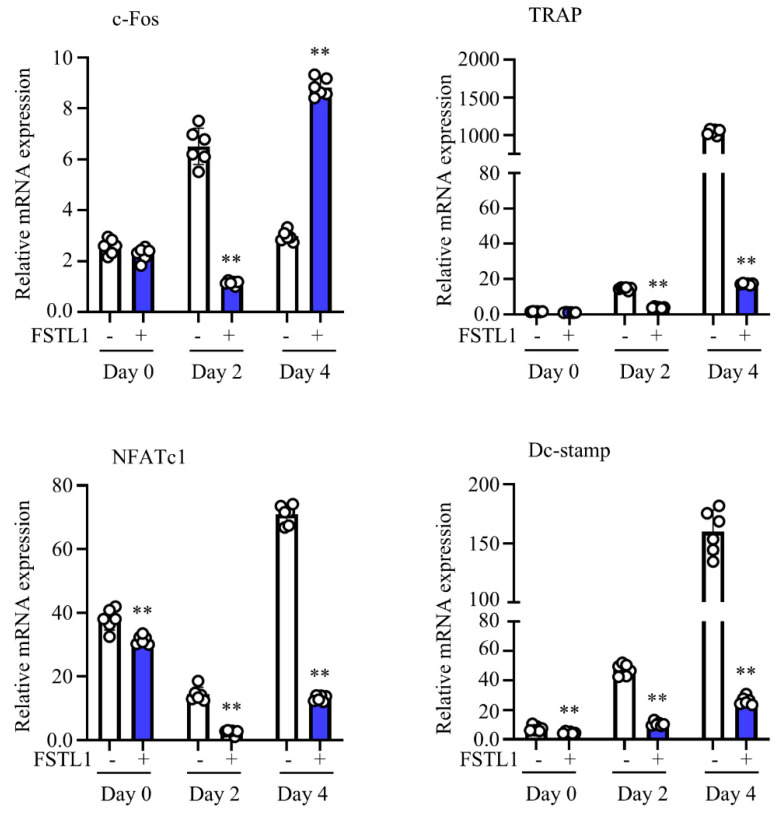
FSTL1 regulates the expression of osteoclastogenic genes. Expression levels of osteoclastogenic markers (c-Fos, TRAP, NFATc1, and DC-STAMP) were analyzed by qRT-PCR during osteoclast differentiation. *n* = 6/group ** *p* < 0.01.

qRT-PCR analysis revealed that rmFSTL1 significantly downregulated TRAP, NFATc1, and DC-STAMP expression during osteoclastogenesis. The early marker c-Fos decreased on day 2 but increased on day 4, indicating a delay in differentiation (Figure 2). Collectively, these results demonstrate that FSTL1 inhibits osteoclast differentiation by modulating key transcriptional regulators.

### 3.3. FSTL1 Stimulated Mesenchymal Cell Chemotaxis and Osteogenic Differentiation

**Figure 3:**
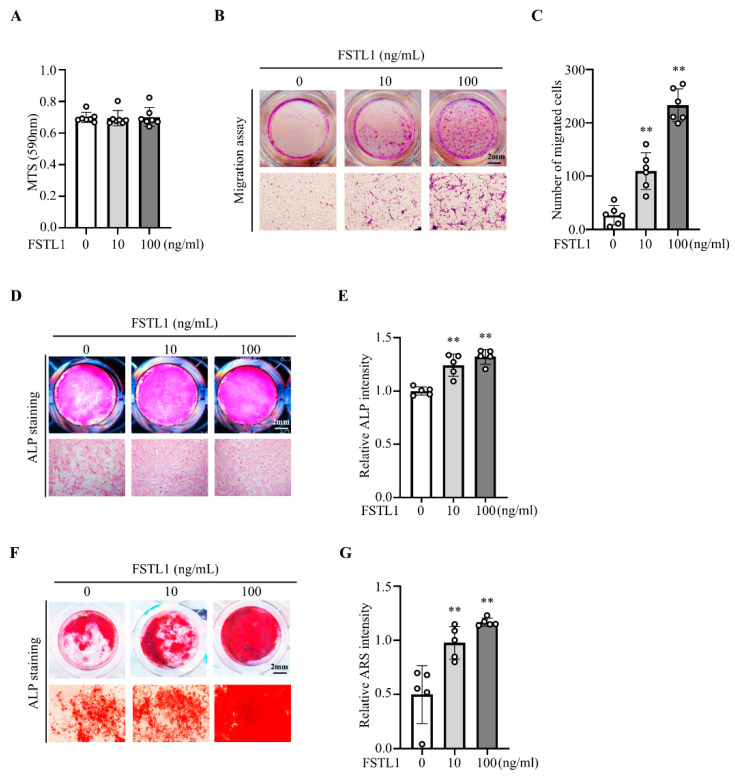
FSTL1 promotes BMSC migration and osteogenic differentiation. (**A**) BMSCs were seeded at 5 × 10^3^ cells/well in 96-well plates, treated with FSTL1 (0, 10, or 100 ng/mL), and proliferation was measured after 24 h using the MTS assay (*n* = 7/group). (**B**) BMSC migratory capacity was evaluated using Transwell chambers. The lower chambers contained α-MEM with 10% FBS and FSTL1 (0, 10, or 100 ng/mL) as a chemoattractant. Migrated cells on the lower surface were stained with crystal violet and imaged using a Leica DMI4000 B microscope (*n* = 5/group). (**C**) The number of migrated cells was quantified using ImageJ software (*n* = 5/group). (**D**) Osteogenic differentiation was induced with osteogenic medium, as shown in the Materials and Methods Section. Early differentiation was assessed by ALP staining on day 7 (*n* = 5/group). (**E**) ALP staining intensity was quantified using Bioquant Osteo 2019 (v19.9.60) (*n* = 5/group). (**F**) Matrix mineralization was evaluated by ARS staining on day 28 (*n* = 5/group). (**G**) The mineralized area was quantified using Bioquant Osteo 2019 (v19.9.60) (*n* = 5/group). ** *p* < 0.01 compared with the control group.

BMSCs were examined to assess the pro-osteogenic effects of FSTL1. MTS assay showed no significant change in proliferation (Figure 3A). In contrast, Transwell migration assays demonstrated that FSTL1 significantly enhanced BMSC chemotaxis at both 10 and 100 ng/mL (Figure 3B,C). Furthermore, ALP and Alizarin Red S (ARS) staining revealed increased early osteogenic activity and enhanced mineralization (Figure 3D–G). These findings establish FSTL1 as a stimulator of mesenchymal cell recruitment and osteogenic differentiation.

### 3.4. FSTL1-Induced Osteoblast Mineralization

**Figure 4:**
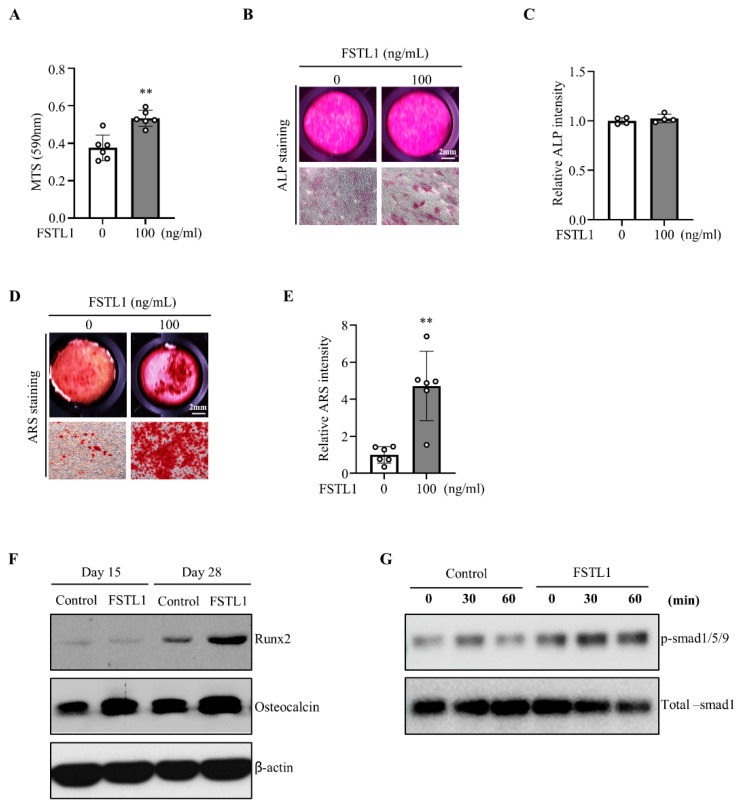
Enhanced osteoblast mineralization under rmFSTL1 treatment. (**A**) Osteoblast proliferation was assessed by MTS assay. Cells were seeded at 5 × 10^3^ cells/well in 96-well plates and treated with FSTL1 (0 or 100 ng/mL) for 24 h (*n* = 6/group). (**B**) Osteoblast differentiation of MC3T3-E1 cells was induced using osteogenic medium. Early differentiation was assessed by ALP staining on day 7 (*n* = 4/group). (**C**) ALP staining intensity was quantified using Bioquant Osteo 2019 (v19.9.60) (*n* = 4/group). (**D**) Osteoblast mineralization was evaluated by ARS staining on day 28 (*n* = 7/group). (**E**) The mineralized area was quantified using Bioquant Osteo 2019 (v19.9.60). (**F**) Protein expression of Runx2 and osteocalcin was analyzed by Western blotting in MC3T3-E1 cells treated with FSTL1 (0 or 100 ng/mL). (**G**) Protein expression of p-Smad1/5/9 and total Smad1 was analyzed by Western blotting in MC3T3-E1 cells treated with FSTL1 (0 or 100 ng/mL). ** *p* < 0.01 compared with the control group.

FSTL1 promoted osteoblast proliferation; however, ALP staining demonstrated only a modest increase, without a statistically significant effect on early osteogenic differentiation (Figure 4A–C). In contrast, ARS staining revealed robust stimulation of mineralization (Figure 4D,E). In the Western blot analysis, the expression levels of Runx2 and osteocalcin were markedly increased on day 28, whereas no significant changes were observed on day 15 (Figure 4F). In addition, to evaluate BMP2 pathway activation, we assessed p-Smad1/5/9 expression following FSTL1 treatment. FSTL1 significantly enhanced p-Smad1/5/9 expression, while total Smad1 levels remained unchanged (Figure 4G). Collectively, these findings demonstrate that FSTL1 promotes osteoblast proliferation and late-stage mineralization, highlighting its role as a positive regulator of bone remodeling.

## 4. Discussion

**Figure 5:**
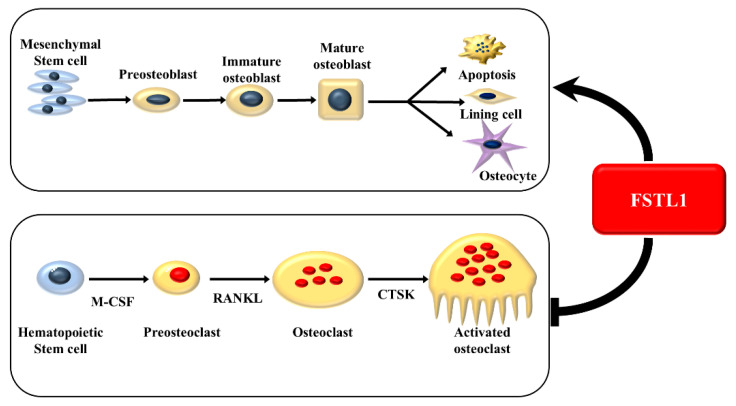
The role of follistatin-like 1 in the cross-talk among osteoclastogenesis, mesenchymal stem cell migration, and osteoblastogenesis. FSTL1 inhibits osteoclast differentiation and resorptive activity, while it promotes mesenchymal cell chemotaxis, osteoblast proliferation, and differentiation.

Bone remodeling requires precise coupling between osteoclast-mediated resorption and osteoblast-mediated formation. Osteoclasts are not only resorptive cells but also active secretory cells that release coupling factors to recruit osteoprogenitors and coordinate new bone formation at resorption sites [1,3,7,9]. In this study, we demonstrate that FSTL1 suppresses osteoclastogenesis and resorptive activity while promoting mesenchymal cell chemotaxis, osteogenic maturation, and osteoblast differentiation under *in vitro* conditions. These findings suggest that FSTL1 contributes to the regulation of osteoclast-osteoblast cellular interactions and may play a role in coordinating bone resorption and formation at the cellular level. Further *in vivo* studies are required to determine its physiological relevance and therapeutic potential in disorders characterized by dysregulated bone remodeling, including osteoporosis (Figure 5).

Our study demonstrates that recombinant FSTL1 inhibited osteoclast differentiation and resorptive function, as evidenced by reduced TRAP staining, diminished pit resorption, and downregulation of osteoclastogenic markers, including TRAP, NFATc1, and DC-STAMP. These findings contrast with a previous report suggesting that FSTL1 promotes osteoclast formation through RANKL-mediated NF-κB activation and M-CSF–induced precursor proliferation [21]. Notably, substantial methodological differences exist between the two studies. In the previous report, BMMs were cultured using CMG-conditioned medium as a source of M-CSF, which contains additional secreted factors derived from the stable producer cell line that may influence osteoclast differentiation independently of the experimental treatment. In contrast, our experiments used recombinant M-CSF alone to precisely control osteoclastogenic signaling. Indeed, for macrophage differentiation, there was a difference in efficacy between recombinant M-CSF and conditioned medium from L929 cells [27]. This distinction in culture conditions likely accounts, at least in part, for the divergent effects of FSTL1 observed between the studies, highlighting the context-dependent role of FSTL1 in osteoclast regulation.

Moreover, this study showed a reduction in c-Fos expression on day 2, which suggests an early suppression of osteoclast commitment. Although c-Fos expression increased on day 4, this late-stage upregulation did not restore osteoclastogenesis, likely because early signaling events required for full differentiation had already been attenuated. Osteoclast formation depends on the coordinated activation of downstream regulators such as NFATc1 and sustained RANKL signaling; thus, delayed or transient c-Fos induction alone may be insufficient to drive normal osteoclast maturation. From a therapeutic standpoint, factors that attenuate osteoclast function while enhancing osteoblast activity are particularly promising, given that many of the currently available anti-resorptive drugs reduce overall bone turnover and may consequently limit new bone formation. In addition to its regulatory effects on osteoclasts, FSTL1 directly targets osteoprogenitors, significantly enhances BMSC chemotaxis, and promotes osteogenic differentiation and mineralization. These findings are consistent with features attributed to the factors involved in osteoclast–osteoblast cellular interactions, particularly in facilitating osteoprogenitor recruitment and differentiation during the transition from resorption to formation. However, further *in vivo* studies are required to confirm its role in physiological bone remodeling [12,14].

Although a previous study reported that FSTL1 suppresses osteoblast differentiation in mesenchymal stem cells [28], the reported study did not observe a significant reduction in ARS staining intensity in FSTL1-treated groups. Moreover, extensive cell loss was evident across all ARS-stained groups, which may have compromised the integrity of the mineralization process and confounded the interpretation of osteogenic outcomes. Taken together, our results suggest that FSTL1 may not only accelerate early osteogenic commitment but also support later maturation toward an osteocyte-like phenotype, a process essential for forming a functional mineralized matrix.

In MC3T3-E1 osteoblasts, FSTL1 increased proliferation and late-stage mineralization, while early differentiation, assessed by ALP staining, remained unchanged. This pattern suggests that FSTL1 preferentially promotes late osteoblast maturation and matrix mineral deposition rather than accelerating early ALP-associated phases. Consistent with this interpretation, FSTL1 induced the expression of Runx2 and osteocalcin, key regulators of lineage commitment and late differentiation. FSTL1 treatment significantly increased Runx2 expression at the late stage of osteoblast differentiation. Runx2 primarily regulates early osteoblast commitment and differentiation [29]. While Runx2 overexpression *in vivo* has been reported to inhibit terminal mineralization [30], other reports indicate that Runx2 may also contribute to osteoblast maturation and mineralization in a context-dependent manner [31]. Therefore, the role of Runx2 in late-stage osteoblast differentiation and mineralization may vary depending on differences between *in vivo* and *in vitro* systems and microenvironment conditions. Together, these findings indicate that FSTL1 contributes to bone formation by (i) recruiting mesenchymal stem cells and (ii) promoting osteogenic maturation, and that future work will be required to quantify their relative contributions *in vivo*.

Mechanistically, the receptor and signaling pathways mediating FSTL1 function in bones remain incompletely defined. FSTL1 has been implicated in diverse biological contexts, including inflammation, tissue repair, fibrosis, and cardiovascular remodeling [18,19]. In cartilage, FSTL1 contributes to extracellular matrix integrity and chondrocyte differentiation, with altered expression reported in osteoarthritic tissues [20]. In bones, our results demonstrate that FSTL1 regulates bone remodeling by inhibiting osteoclast activity and differentiation while promoting osteoprogenitor recruitment, osteogenic differentiation, and maturation.

This study has several limitations. First, our conclusions are based on *in vitro* systems (BMM-derived osteoclasts, primary BMSCs, and MC3T3-E1 cells), and *in vivo* validation will be necessary to confirm the role of FSTL1 during physiological remodeling or in disease contexts, such as ovariectomy-induced osteoporosis. Second, the receptor and downstream signaling mechanism remain undefined. Third, early osteogenic differences between BMSCs and MC3T3-E1 cells suggest that cell context strongly influences FSTL1 responsiveness. Despite these limitations, our findings support a conceptual framework in which osteoclast-derived FSTL1 contributes to the resorption-to-formation transition by coordinating both arms of the remodeling process.

## 5. Conclusions

In summary, FSTL1 suppresses osteoclast differentiation and resorptive activity while enhancing mesenchymal cell chemotaxis and osteogenic maturation. It promoted BMSC migration, increased the expression of osteogenic markers and mineralization, and enhanced MC3T3-E1 osteoblast proliferation and mineralization, accompanied by the induction of Runx2 and osteocalcin. Collectively, these findings indicate that FSTL1 regulates both osteoclast and osteoblast lineage activities under *in vitro* conditions, influencing processes related to bone resorption and formation at the cellular level. These results suggest that FSTL1 potentially plays a role in the coordination of bone remodeling; however, further *in vivo* studies are required to establish its physiological relevance and therapeutic potential in osteoporosis and other disorders characterized by impaired bone remodeling.

## Figures and Tables

**Table 1:** Primer sequences for qRT-PCR.

Target Gene	Forward Sequence (5′–3′)	Reverse Sequence (5′–3′)
*Gapdh*	GCA TCT CCC TCA CAA TTT CCA	GTG CAG CGA ACT TTA TTG ATG G
*c-Fos*	AGG CCC AGT GGC TCA GAG A	GCT CCC AGT CTG CTG CAT AGA
*TRAP*	TCC CCA ATG CCC CAT TC	CGG TTC TGG CGA TCT CTT TG
*NFATc1*	ACC ACC TTT CCG CAA CCA	TTC CGT TTC CCG TTG CA
*DC-STAMP*	CTT CCG TGG GCC AGA AGT T	AGG CCA GTG CTG ACT AGG ATG A

## Data Availability

The original contributions presented in this study are included in the article. Further inquiries can be directed to the corresponding author(s).
